# A 2023 nationwide study on adjustment disorder among first year MBBS students in India

**DOI:** 10.6026/973206300200190

**Published:** 2024-02-29

**Authors:** Mayank Agarwal, Priyanka Sharma, Ayan Goswami, Roopali Mittal

**Affiliations:** 1 Department of Physiology, All India Institute of Medical Sciences, Raebareli, Uttar Pradesh, India; 2 Department of Physiology, School of Medical Sciences and Research, Sharda University, Greater Noida, India; 3 Department of Physiology, Santiniketan Medical College, Bolpur, West Bengal, India

**Keywords:** Adjustment disorder, India, medical students, mental health, undergraduate medical education

## Abstract

Amidst the complex transition to medical college, encompassing a myriad of academic, social, and personal adjustments, MBBS students in India confront multifaceted challenges that can precipitate adjustment disorder, a phenomenon understudied within the Indian context. Therefore, it is of interest to assess adjustment disorder among first-year MBBS students within six months of enrolment. We used a Google form encompassing adjustment disorder new-module 20 for data collection and found that 67% of the 401 responses from first-year medical students exhibited adjustment disorder. The top stressors identified included time pressure, work pressure, financial problems, moving to a new home, and termination of important leisure activities. Female gender, age group 21-25, conflicts in working life, financial problems, own serious illness, family conflicts, pressure to meet deadlines, and excessive workload showed significant associations with adjustment disorder.

## Background:

First-year Bachelor of Medicine and Bachelor of Surgery (MBBS) students undergo significant social and psychological changes as they transition from school to medical college [1,2]. Challenges in academic life are often complex, encompassing various factors that include but are not limited to personal, social, financial, and health aspects [2,3,4, 5,6]. These challenges, which can arise at any point, are particularly pronounced for first-year students who encounter issues like homesickness and social stress due to communication barriers, gender-related factors, socioeconomic status, disorientation, culture shock, and varying educational expectations [7]. Medical education requires a faster, continuous, and self-directed learning pace compared to intermediate schooling, where students must reflect on their progress, identify knowledge and skill gaps, and actively seek information to address them, amplifying stress and anxiety during the commencement of medical training if they feel unprepared [8]. Enrolling in medical college can be a significant stressor, leading to various emotional and psychological challenges, as indicated by a study stating that about 50% of newly enrolled university students experience academic adjustment problems, which are significantly associated with Adjustment Disorder (AjD) [9]. A study conducted in South India revealed that 48% of medical students had low resilience to stress, and 52% displayed low coping abilities [10]. The American Psychiatric Association's Diagnostic and Statistical Manual of Mental Disorders - fifth edition (DSM-5) defines Adjustment Disorder (AjD) as the manifestation of emotional or behavioral symptoms triggered by an identifiable stressor, typically emerging within three months of the stressor's onset and not persisting for more than six months after its resolution [11]. The International Statistical Classification of Diseases and Related Health Problems, eleventh edition (ICD-11), describes AjD as a maladaptive reaction typically occurring within a month of a significant life stressor [11]. The Adjustment Disorder-New Module 20 (ADNM-20) is a reliable and valid scale that conveniently assesses AjD [12- 13]. Despite reports of heightened psychological distress among medical students in India [14], there is a noticeable research gap concerning the impact of distress on the student's psyche that could potentially contribute to the development of AjD. Two large-scale surveys from North and South India in 2019 and South and East India in 2020 revealed that around 80% of Indian medical students experienced burnout [15, 16]. However, these studies did not specifically target first-year students. Additionally, one of the studies was conducted during the coronavirus disease 2019 (COVID-19) pandemic [16]. Therefore, it is of interest to report a 2023 nationwide study on adjustment disorder among first year MBBS students in India.

## Materials and Methods:

This web-based observational cross-sectional study was conducted from February 2023 to April 2023. Ethical approval (SMC/IEC/17012023) was obtained from the Institutional Ethics Committee of Santiniketan Medical College, Bolpur, West Bengal. The inclusion criteria comprised first-year MBBS students at Indian medical colleges who had experienced stress within six months of enrolment, were 18 years and above, and were willing to participate. Based on India's more than one lakh MBBS seats, we utilized the Cochran formula to determine the sample size. For a population size exceeding one lakh, with a 95% confidence interval (CI) and a 5% margin of error, the estimated sample size was determined to be 383. Data were collected through a Google form that included details of informed consent, demographic information, and the ADNM-20 questionnaire. The first part of the ADNM-20 questionnaire presented respondents with a list of sixteen stressors and an option to mention any other recent stressors. The second part consisted of a twenty-item list assessing symptoms associated with AjD in the past six months. Respondents used a four-point Likert scale to express the frequency of these symptoms: one implied never, two indicated rarely, three denoted sometimes, and four represented often. These twenty items were categorized into six subscales: preoccupation with the stressor/s, failure to adapt, avoidance, depressed mood, anxiety, and impulse disturbance. The sum score of all items exceeding 48 was used as the cut-off value (87% sensitivity, 74% specificity, 57% positive predictive value, and 93% negative predictive value) for the diagnosis of AjD [17].

## Statistical analysis:

The data from the Google form was imported into Microsoft Excel 365, and subsequent statistical analysis was conducted using IBM SPSS Statistics for Windows, Version 27. The data were presented as percentages (rounded to one decimal place), frequencies, and medians with interquartile range (IQR). Multiple response dichotomy analysis was applied to calculate the frequency of stressors in percentage. The Mann-Whitney U test was used to compare the ADNM-20 score and its subscale scores among participants with and without AjD, males and females, and participants under 21 years and those aged 21-25. The chi-square test was used to assess the association of categorical variables. Multivariate binomial logistic regression analysis evaluated the multivariate Adjusted Odds Ratio (AOR) with a 95% CI. Statistical significance was considered at P ≤ 0.05.

## Results:

The demographic information of the 401 respondents is summarized in Table 1. Responses were received from two age groups, with participants falling into the categories of less than 21 years old (but not less than 18 years old) and 21 to 25 years old. All participants were unmarried and not diagnosed with any mental disorder before entering medical college. Most responses (93.8%) were from India's North and East zones. The list of stressors provided a multiple-response and semi-closed question format, allowing respondents to select from sixteen listed stressors or mention any other recent stressors faced after entering medical college. Following data cleaning, responses regarding 'any other stressful event' were categorized into 'stress or depression' and 'personal life conflict' were collected. Table 2 presents the frequency of stressors chosen and mentioned by respondents. After adjusting for gender and other stressors, multivariate binomial regression analysis revealed a significant (P = 0.029) association (AOR = 1.7, 95% CI: 1.1-2.7) between the 'too much or too little' stressor with the <21 age group, while 'stress or depression' mentioned by respondents was significantly (P = 0.017) associated (AOR = 13.2, 95% CI: 1.6-108.8) with the 21-25 age group. Gender was not significantly associated with stressors. ADNM-20 score analysis shows that 268 (67%) respondents had scores exceeding 48, indicating the presence of AjD. The highest possible score for ADNM-20 is 80, while the lowest is 20. Table 4 displays the ADNM-20 median (IQR) scores for all respondents, males, females, <21 years old, and 21-25 years old respondents. ADNM-20 scores were significantly higher for females compared to males and for the 21-25 age group compared to the <21 age group. Table 3 shows that all six subscales of the ADNM-20 were significantly higher in respondents with AjD than those without AjD. Similarly, all six subscales were significantly higher for the 21-25 age group than the <21 age group. Further, except for 'failure to adapt,' all other five subscales were significantly higher for females than males. The highest possible score for preoccupation with stressors, failure to adapt, and avoidance is sixteen, while the lowest is four. For depressed mood and impulse disturbance, the highest possible score is twelve, while the lowest is three. The highest possible score for anxiety is eight, while the lowest is two. Table 4 presents the results of multivariate binomial regression analysis, indicating that females and the 21-25 age group were at significantly higher risk of developing AjD than males and the <21 age group. Additionally, 'family conflicts,' 'conflicts in working life,' 'too much or too little work,' 'pressure to meet deadlines or time pressure,' 'financial problems,' and 'own serious illness' were significantly associated with the development of AjD among the listed and mentioned stressors.

## Discussion:

This cross-sectional web-based study aimed to assess AjD among first-year MBBS students in Indian medical colleges within the last six months and explored related risk factors. Results showed that two-thirds of students experiencing stressors suffered from AjD, with females and the 21-25 age group being more susceptible than males and the 18-20 age group. Makki *et al.* [5] discovered AjD in 47% of first- and second-year medical students in two Saudi Arabian medical colleges, while Alhussain *et al.* [4] found AjD in 55% of first-year medical students facing stressors in Saudi Arabia. In contrast, our study revealed a higher proportion (67%) of AjD among first-year MBBS students compared to previous research. Two studies from Ethiopia reported a prevalence of approximately 42% for adjustment problems among first-year undergraduate students at different Ethiopian universities [7, 18]. However, it is imperative to highlight that medical students experience heightened psychological distress compared to peers of similar age despite comparable or healthier backgrounds [6], suggesting that the medical education process itself may contribute to student distress. A study from South India further supports this notion, highlighting medical education as an unavoidable stressor for first-year MBBS students [19].

Students with AjD exhibited significantly higher scores across all six subscales of ADNM-20 compared to those without AjD, with the 21-25 age group displaying more pronounced symptoms than the 18-20 age group. Consistent with our results, a study from South India found a significant association between stress and medical students over 20 years of age compared to those less than 20 years of age [20]. In contrast, Alhussain *et al.* noted that younger students were more susceptible to AjD [4]. It is important to note that the >21 age group constituted only 4% in Alhussain *et al.*'s study, while in our study, the 21-25 age group comprised 37% of respondents. Contrary to our findings, Alnakhli *et al.* found that the 18-20 age group was more susceptible to AjD than the 21-24 age group among medical students at Arabian Gulf University in Bahrain [21]. Age was not associated with AjD in Makki *et al.*'s findings [5].

Except for the failure to adapt subscale, females demonstrated significantly more profound symptoms of AjD than males, aligning with Graves *et al.*'s study, which found that female undergraduates experience higher stress than males [22]. Additionally, consistent with our results, Alhussain *et al.* and Alnakhli *et al.* reported that females were at a higher risk than males for developing AjD [4, 21]. Gender was not associated with AjD in Makki *et al.*'s findings [5]. In our study, the majority of students (60%) experienced academic-life-related stressors, which included time pressure, work pressure, moving to a new home, and decreased leisure time. Similar findings were reported in a study from East India, where 79% of first-year MBBS students had high to severe levels of academic stress [23], and in another study from North India, where first-year MBBS students faced higher stress related to academic aspects than social stress [24]. The 18-20 age groups was significantly more affected by work pressure than the 21-25 age group. In addition, eight respondents in the 21-25 age group mentioned being under stress or depression, apart from choosing other stressors. Frequencies of stressors were similar between males and females.

Our study suggests that time pressure, work pressure, conflicts in medical college, and financial problems are significant independent risk factors for AjD in first-year MBBS students. Alhussain *et al.* also reported that work pressure in first-year MBBS students is significantly associated with a higher risk of AjD development [4]. Work pressure, time pressure, and financial problems also emerged as the top three stressors among first- and second-year medical students in Saudi Arabia [5]. Similarly, Hill *et al.* reported academic workload, time constraints, and financial concerns as the most mentioned stressors by medical students [6]. Personal issues like family conflict, illness of a loved one, death of a loved one, and own serious illness are also essential factors in the development of AjD. In our study, own serious illness and family conflicts were independently associated with AjD. However, in contrast to our findings, Aziz *et al.* reported that medical students face more significant emotional and family-related issues and fewer academic-related problems [25]. It is important to note that the study by Aziz *et al.* included second to fifth-year students and did not involve first-year students.

Adjustment is crucial for managing stress and enhancing academic performance. However, our results indicate that many students struggle to adapt to the demands of the medical curriculum, leading to AjD. Therefore, medical colleges must assist students in navigating and adapting to these demands, supporting the need for mental health wellness interventions and stress reduction initiatives to maximize resilience to AjD. Introducing mindfulness-based interventions or psychological skills laboratories in the first-year curriculum could effectively address AjD [26,27].

## Limitations:

The study's cross-sectional design prevents us from establishing a causal relationship between the observed associations. Additionally, recall bias was inevitable since the data relied on self-reports from participants. Furthermore, our examination focused solely on stressors experienced by first-year MBBS students within the initial six months of enrolment in medical college, with responses predominantly originating from India's North and East regions.

## Conclusion:

Data shows that two-thirds of students experiencing recent stressors after entering medical college developed AjD, with females and individuals aged 21-25 years facing a higher likelihood of AjD compared to males and those aged 18-20 years. Academic life-related stressors were more prevalent than health-related, social, and interpersonal stressors in AjD development, with conflict in working life and financial burden emerging as the most substantial risk factors. The notable influence of academic-life-related stressors underscores the urgent need for customized mental health interventions and stress management programs within medical education.

## Figures and Tables

**Table 1:** The demographic information of the respondents

**Demographic characteristic (n=401)**	**Frequency (%)**
Age group	
<21 years (18-20 years)	251 (62.6%)
21-25 years	150 (37.4%)
Gender	
Male	207 (51.6%)
Female	194 (48.4%)
Marital status	
Married	0 (0%)
Unmarried	401 (100%)
Prior mental disorder	
Have you ever been diagnosed with a mental disorder prior to joining the medical institute?	No (100%)
Location of respondent's medical college	
North zone (268, 66.8%)	Chandigarh (2)
	Delhi (46)
	Haryana (64)
	Himachal Pradesh (1)
	Punjab (1)
	Jammu and Kashmir (3)
	Rajasthan (39)
	Uttarakhand (1)
	Uttar Pradesh (111)
Northeast zone (7, 1.8%)	Assam (4)
	Tripura (3)
East zone (109, 27.2%)	Bihar (33)
	Jharkhand (5)
	Odisha (2)
	West Bengal (69)
South zone (3, 0.7%)	Karnataka (1)
	Kerala (1)
	Puducherry (1)
West zone (9, 2.2%):	Maharashtra (9)
Central zone (5, 1.3%):	Madhya Pradesh (5)

**Table 2:** List of stressors along with their frequencies

**Stressors**		**Frequency of responses**				
		**Overall (n=401)**	**Male (n=207)**	**Female (n=194)**	**<21 years (n=251)**	**21-25 years (n=150)**
Pressure to meet deadlines/time pressure		164 (24.0%)	82 (22.7%)	82 (25.4%)	100 (23.3%)	64 (25.2%)
Too much / too little work		160 (23.4%)	82 (22.7%)	78 (24.1%)	112* (26.0%)	48* (18.9%)
Financial problems		89 (13.0%)	48 (13.3%)	41 (12.7%)	50 (11.6%)	39 (15.4%)
Moving to a new home		51 (7.5%)	24 (6.6%)	27 (8.4%)	33 (7.7%)	18 (7.1%)
Termination of an important leisure activity		36 (5.3%)	20 (5.5%)	16 (5.0%)	22 (5.1%)	14 (5.5%)
Family conflicts		32 (4.7%)	14 (3.9%)	18 (5.6%)	20 (4.7%)	12 (4.7%)
Conflicts in working life		27 (3.9%)	14 (3.9%)	13 (4.0%)	16 (3.7%)	11 (4.3%)
Death of a loved one		24 (3.5%)	11 (3.0%)	13 (4.0%)	12 (2.8%)	12 (4.7%)
Own serious illness		24 (3.5%)	14 (3.9%)	10 (3.1%)	15 (3.5%)	9 (3.5%)
Unemployment		19 (2.8%)	11 (3.0%)	8 (2.5%)	14 (3.3%)	5 (2.0%)
Illness of a loved one		14 (2.0%)	10 (2.8%)	4 (1.2%)	10 (2.3%)	4 (1.6%)
Conflicts with neighbours		10 (1.5%)	6 (1.7%)	4 (1.2%)	7 (1.6%)	3 (1.2%)
Assault		10 (1.5%)	6 (1.7%)	4 (1.2%)	7 (1.6%)	3 (1.2%)
Any other stressful event	a. Stress or depression	9 (1.3%)	6 (1.7%)	3 (0.9%)	1* (0.2%)	8* (3.1%)
	b. Personal life conflict	6 (0.9%)	5 (1.4%)	1 (0.3%)	5 (1.2%)	1 (0.4%)
Divorce/separation		5 (0.7%)	4 (1.1%)	1 (0.3%)	2 (0.5%)	3 (1.2%)
Serious accident		4 (0.6%)	4 (1.1%)	0 (0%)	4 (0.9%)	0 (0%)
Adjustment due to retirement		0 (0%)	0 (0%)	0 (0%)	0 (0%)	0 (0%)
Total		684	361	323	430	254

**Table 3:** The median (IQR) scores for the ADNM-20 scale and its subscales for all respondents, those with and without adjustment disorder, males, females, less than 21, and 21-25 age group respondents

**Parameter**	**Overall (n=401)**	**With adjustment disorder (n=268)**	**Without adjustment disorder (n=133)**	**P**	**Male (n=207)**	**Female (n=194)**	**P**	**<21 years (n=251)**	**21-25 years (n=150)**	**P**
ADNM-20	56 (44-62)	60 (56-65)	40 (31-44)	<0.001	52 (41-61)	58 (48-64)	<0.001	53 (42-61)	60 (47-65)	<0.001
Preoccupation with stressor	11 (8.5-13)	12 (11-14)	7 (5-9)	<0.001	11 (7-12)	12 (9-13.25)	<0.001	11 (8-13)	12 (9-14)	0.017
Failure to adapt	10 (7-12)	12 (10-13)	6 (4-8)	<0.001	10 (7-12)	10.5 (8-12)	0.071	9 (7-12)	11 (8-13)	<0.001
Avoidance	12 (10-14)	13 (12-14)	9 (7-11)	<0.001	12 (9-13)	13 (11-14)	<0.001	12 (10-13)	13 (10-14)	<0.001
Depressed mood	8 (7-9)	9 (8-10)	6 (5-7)	<0.001	8 (6-9)	9 (7-10)	<0.001	8 (7-9)	9 (7-10)	0.013
Anxiety	6 (4-7)	6 (6-7)	4 (2-5)	<0.001	5 (4-6)	6 (5-7)	0.001	6 (4-6)	6 (5-7)	<0.001
Impulse disturbance	8 (6-9)	9 (8-10)	5 (3-6)	<0.001	7 (5-9)	9 (6.25-10)	<0.001	7 (5-9)	9 (6-10)	<0.001

**Table 4:** Statistically significant demographic characteristics and stressors associated with adjustment disorder.

**Characteristic**	**Adjustment disorder present**		**AOR (95% CI)**	**P**
	**Yes**	**No**		
Age-group				
<21 years	157 (63%)	94 (37%)	1.9 (1.1-3.2)	0.024
21-25 years	111 (74%)	39 (26%)		
Gender				
Male	119 (57%)	88 (43%)	2.8 (1.7-4.7)	<0.001
Female	149 (77%)	45 (23%)		
Family conflicts				
Yes	26 (81%)	6 (9%)	3.6 (1.2-10.6)	0.024
No	242 (66%)	127 (34%)		
Conflicts in working life				
Yes	26 (96%)	1 (4%)	15.0 (1.8-126.6)	0.013
No	242 (65%)	132 (35%)		
Too much/ too little work				
Yes	115 (72%)	45 (28%)	3.0 (1.6-5.5)	<0.001
No	153 (63%)	88 (37%)		
Pressure to meet deadlines/ time pressure				
Yes	127 (77%)	37 (23%)	3.3 (1.8-5.8)	<0.001
No	141 (60%)	96 (40%)		
Financial problems				
Yes	79 (89%)	10 (11%)	8.2 (3.5-19.4)	<0.001
No	189 (61%)	123 (39%)		
Own serious illness				
Yes	21 (88%)	3 (12%)	5.7 (1.4-23.1)	0.015
No	247 (66%)	130 (34%)		
